# Practical testing of Scots pine cutting propagation - a joint Metla-Skogforsk-Silava project

**DOI:** 10.1186/1753-6561-5-S7-P129

**Published:** 2011-09-13

**Authors:** Karl-Anders Högberg, Jörgen Hajek, Arnis Gailis, Niina Stenvall, Inga Zarina, Satu Teivonen, Tuija Aronen

**Affiliations:** 1 Skogforsk, Ekebo, Sweden; 2 Skogforsk, Sävar, Sweden; 3 Silava, Latvia; 4 Finnish Forest Research Institute, Haapastensyrjä, Finland; 5 Finnish Forest Research Institute, Punkaharju, Finland

## 

Testing of candidates as clones would greatly benefit breeding of Scots pine (*Pinus sylvestris* L.), but has not been applied because of vegetative propgation of the species is difficult. With a common interest in Scots pine breeding, forest research institutions from Sweden, Finland, and Latvia (Skogforsk, Metla, and Silava, respectively) joined in a collaborative project to develop pine cutting propagation for breeding purposes. The main objective of this effort was to find protocols for sufficient shoot production, and at the same time, maintain a high rooting response. Secondly, the aim was to increase the knowledge on the influence of different rooting agents, watering regimes and substrates on the rooting of cuttings.

From Finland and Latvia, respectively, 15 full-sib families from parents with high breeding values were included. From Sweden, 15 families from each of two research stations, one northern and one southern, were included. These sets of the families were used locally. Another collection of five families was shared among the participants, which means that 5 families extra were included at each location.All donor plants were pruned according to the same principles. However, the timing of the pruning varied in the five propagation models tested:

A. One-year-old donor plants pruned to yield 2x winter cuttings / 4-year method

B. One-year-old donor plants pruned to yield winter cuttings and late summer cuttings/ 3-year method

C. “Turbo line” i.e. one-year-old donor plants pruned 2 x cuttings in one year/ 2-year method

D.One-year-old donor plants pruned to yield 2x late summer cuttings/ 3-year method

E. Two-year-old donor plants pruned to yield winter cuttings/ 4-year method

According to the results, the average production of cuttings with propagation models including two harvests in consecutive years on the same donor plant can be predicted to be 10-15 cuttings per a donor plant, with a substantial variation among families and donor plants. Even though rooting responses above 50 % can be achieved, this could not be repeated for a large number of propagations. The generally low and erratic rooting response leads to conclusion that the project was unsuccessful in developing improved and reliable protocols for Scots pine cutting propagation. However, a propagation model enabling two harvests on a 1-year-old donor plant within the same year is a promising option but needs further studies.

More detailed information on the project and its results is available at: http://www.metla.fi/julkaisut/workingpapers/2011/mwp198.htm

